# Supplementary material for: Generalisable deep learning method for mammographic density prediction across imaging techniques and self-reported race
Source: Commun Med (Lond). 2024 Feb 19;4:21. doi: 10.1038/s43856-024-00446-6 (PMC10876691; doi:10.1038/s43856-024-00446-6)
Supplement: Supplementary file 1 — Description of Additional Supplementary Files [file 43856_2024_446_MOESM1_ESM.docx]

**Description of Additional Supplementary Files**

**File Name:** Supplementary Data

**Description:** Supplementary Data contains the source data for generating Figures 2 and 3.
