# Supplementary material for: Generalisable deep learning method for mammographic density prediction across imaging techniques and self-reported race
Source: Commun Med (Lond). 2024 Feb 19;4:21. doi: 10.1038/s43856-024-00446-6 (PMC10876691; doi:10.1038/s43856-024-00446-6)
Supplement: Supplementary file 2 — Supplementary Information [file 43856_2024_446_MOESM2_ESM.pdf]

Generalisable deep learning method for mammographic density prediction across imaging techniques and self-reported race

Galvin Khara, Hari Trivedi, Mary S. Newell, Ravi Patel, Tobias Rijken, Peter Kecskemethy, Ben Glocker

Supplementary information

Statistical testing of performance equivalence per model across races

Supplementary Table 1. TOST p-value results for model A across races.

Model A	P-values		
Race group	Black	White	Asian
Black	$< 10^{-5}$	$< 10^{-5}$	$< 10^{-5}$
White	$< 10^{-5}$	$< 10^{-5}$	$< 10^{-5}$
Asian	$< 10^{-5}$	$< 10^{-5}$	$< 10^{-5}$

Model A is trained on data from Black participants only. TOST p-value less than 0.05 for all sub-group comparisons at an equivalence margin of 2%.

Supplementary Table 2. TOST p-value results for model B across races.

Model B	P-values		
Race group	Black	White	Asian
Black	$< 10^{-5}$	0.002	$< 10^{-5}$
White	9×10^{-4}	$< 10^{-5}$	$< 10^{-5}$
Asian	$< 10^{-5}$	$< 10^{-5}$	$< 10^{-5}$

Model B is trained on data from White participants only. TOST p-value less than 0.05 for all sub-group comparisons at an equivalence margin of 2%.

Supplementary Table 3. TOST p-value results for model C across races.

Model C	P-values		
Race group	Black	White	Asian
Black	$< 10^{-5}$	$< 10^{-5}$	$< 10^{-5}$
White	$< 10^{-5}$	$< 10^{-5}$	$< 10^{-5}$
Asian	$< 10^{-5}$	$< 10^{-5}$	$< 10^{-5}$

Model C is trained on data from both White and Black participants. TOST p-value less than 0.05 for all sub-group comparisons at an equivalence margin of 2%.

Statistical testing of performance equivalence per race across models

Supplementary Table 4. TOST p-value results for Black participants across models.

Black	P-values		
Model	Model A	Model B	Model C
Model A	$< 10^{-5}$	$< 10^{-5}$	$< 10^{-5}$
Model B	$< 10^{-5}$	$< 10^{-5}$	$< 10^{-5}$
Model C	$< 10^{-5}$	$< 10^{-5}$	$< 10^{-5}$

Model A is trained on data from Black participants only, model B is trained on data from White participants only, and model C is trained on data from both White and Black participants. TOST p-value less than 0.05 for all sub-group comparisons at an equivalence margin of 2%.

Supplementary Table 5. TOST p-value results for White participants across models.

White	P-values		
Model	Model A	Model B	Model C
Model A	$< 10^{-5}$	$< 10^{-5}$	$< 10^{-5}$
Model B	$< 10^{-5}$	$< 10^{-5}$	$< 10^{-5}$
Model C	$< 10^{-5}$	$< 10^{-5}$	$< 10^{-5}$

Model A is trained on data from Black participants only, model B is trained on data from White participants only, and model C is trained on data from both White and Black participants. TOST p-value less than 0.05 for all sub-group comparisons at an equivalence margin of 2%.

Supplementary Table 6. TOST p-value results for Asian participants across models.

Asian	P-values		
Model	Model A	Model B	Model C
Model A	$< 10^{-5}$	2×10^{-5}	$< 10^{-5}$
Model B	3×10^{-4}	$< 10^{-5}$	5×10^{-4}
Model C	$< 10^{-5}$	0.02	$< 10^{-5}$

Model A is trained on data from Black participants only, model B is trained on data from White participants only, and model C is trained on data from both White and Black participants. TOST p-value less than 0.05 for all sub-group comparisons at an equivalence margin of 2%.
