# Supplementary material for: Generalisable deep learning method for mammographic density prediction across imaging techniques and self-reported race
Source: Commun Med (Lond). 2024 Feb 19;4:21. doi: 10.1038/s43856-024-00446-6 (PMC10876691; doi:10.1038/s43856-024-00446-6)
Supplement: Supplementary file 5 — Reporting Summary [file 43856_2024_446_MOESM5_ESM.pdf]

nature portfolio

Corresponding author(s): Ben Glocker, Galvin Khara

Last updated by author(s): Jan 3, 2024

# Reporting Summary

Nature Portfolio wishes to improve the reproducibility of the work that we publish. This form provides structure for consistency and transparency in reporting. For further information on Nature Portfolio policies, see our Editorial Policies and the Editorial Policy Checklist.

## Statistics

For all statistical analyses, confirm that the following items are present in the figure legend, table legend, main text, or Methods section.

| n/a | Confirmed | |
|---|---|---|
| ☐ | ☒ | The exact sample size (*n*) for each experimental group/condition, given as a discrete number and unit of measurement |
| ☐ | ☒ | A statement on whether measurements were taken from distinct samples or whether the same sample was measured repeatedly |
| ☒ | ☐ | The statistical test(s) used AND whether they are one- or two-sided<br>*Only common tests should be described solely by name; describe more complex techniques in the Methods section.* |
| ☐ | ☒ | A description of all covariates tested |
| ☐ | ☒ | A description of any assumptions or corrections, such as tests of normality and adjustment for multiple comparisons |
| ☐ | ☒ | A full description of the statistical parameters including central tendency (e.g. means) or other basic estimates (e.g. regression coefficient) AND variation (e.g. standard deviation) or associated estimates of uncertainty (e.g. confidence intervals) |
| ☐ | ☒ | For null hypothesis testing, the test statistic (e.g. *F*, *t*, *r*) with confidence intervals, effect sizes, degrees of freedom and *P* value noted<br>*Give P values as exact values whenever suitable.* |
| ☒ | ☐ | For Bayesian analysis, information on the choice of priors and Markov chain Monte Carlo settings |
| ☒ | ☐ | For hierarchical and complex designs, identification of the appropriate level for tests and full reporting of outcomes |
| ☒ | ☐ | Estimates of effect sizes (e.g. Cohen's *d*, Pearson's *r*), indicating how they were calculated |

*Our web collection on statistics for biologists contains articles on many of the points above.*

## Software and code

Policy information about availability of computer code

| | |
|---|---|
| Data collection | No data was collected as part of this study. The data used is described and available here: https://github.com/Emory-HITI/EMBED_Open_Data |
| Data analysis | The code used to process the raw data and to develop the deep learning models is integrated within a commercial production system and therefore cannot be released in full. The implementation details provided in the Methods section are sufficient to replicate the deep learning models with open-source frameworks such as TensorFlow or PyTorch. We provide an example implementation on https://github.com/Kheiron-Medical/mammo-net which demonstrates the training and testing of state-of-the-art convolutional neural networks which build the core component of most commercially available breast imaging AI systems. |

For manuscripts utilizing custom algorithms or software that are central to the research but not yet described in published literature, software must be made available to editors and reviewers. We strongly encourage code deposition in a community repository (e.g. GitHub). See the Nature Portfolio guidelines for submitting code & software for further information.

## Data

Policy information about availability of data

All manuscripts must include a data availability statement. This statement should provide the following information, where applicable:

- Accession codes, unique identifiers, or web links for publicly available datasets
- A description of any restrictions on data availability
- For clinical datasets or third party data, please ensure that the statement adheres to our policy

This study made use of a data sample from the EMory BrEast imaging Dataset (EMBED). Access to this data is provided upon request. Contact email: hari.trivedi@emory.edu. More details can be found on https://registry.opendata.aws/emory-breast-imaging-dataset-embed/. Additional information related to this study is available on request to the corresponding author.

## Research involving human participants, their data, or biological material

Policy information about studies with human participants or human data. See also policy information about sex, gender (identity/presentation), and sexual orientation and race, ethnicity and racism.

| | |
|---|---|
| Reporting on sex and gender | All data used was from female participants. |
| Reporting on race, ethnicity, or other socially relevant groupings | The Methods section provides details about the definition of race groups used in the study. |
| Population characteristics | Population characteristics are described in Table 1 in the manuscript and include age, race, and breast density. |
| Recruitment | No participants were recruited. Only previously collected data was used, which is described in detail in https://pubs.rsna.org/doi/10.1148/ryai.220047 |
| Ethics oversight | This research is exempt from ethical approval as the analysis is based on secondary, fully anonymised data which is publicly available. The ethics information about the original data collection is available in https://pubs.rsna.org/doi/10.1148/ryai.220047. |

Note that full information on the approval of the study protocol must also be provided in the manuscript.

# Field-specific reporting

Please select the one below that is the best fit for your research. If you are not sure, read the appropriate sections before making your selection.

☒ Life sciences ☐ Behavioural & social sciences ☐ Ecological, evolutionary & environmental sciences

For a reference copy of the document with all sections, see nature.com/documents/nr-reporting-summary-flat.pdf

# Life sciences study design

All studies must disclose on these points even when the disclosure is negative.

| | |
|---|---|
| Sample size | Largest possible sets were used for training and testing of the machine learning algorithms. The sample corresponds to the largest possible set including all patients that identified as either White, Asian, or Black. |
| Data exclusions | No further data was excluded from the study sample. |
| Replication | The experiments have been run multiple times and no significant differences were found between individual runs. |
| Randomization | The imaging scans are divided randomly into three sets for training (230,954), validation (39,494) and testing (181,194). No scans from the same patient are used in different sets. The validation set is used for model selection, while the test set is the hold out set for measuring the test accuracy of different models. |
| Blinding | Blinding was not relevant in this study as no human intervention was necessary in the data analysis. |

# Reporting for specific materials, systems and methods

We require information from authors about some types of materials, experimental systems and methods used in many studies. Here, indicate whether each material, system or method listed is relevant to your study. If you are not sure if a list item applies to your research, read the appropriate section before selecting a response.

## Materials & experimental systems

| n/a | Involved in the study | |
|---|---|---|
| ☒ | ☐ | Antibodies |
| ☒ | ☐ | Eukaryotic cell lines |
| ☒ | ☐ | Palaeontology and archaeology |
| ☒ | ☐ | Animals and other organisms |
| ☐ | ☒ | Clinical data |
| ☒ | ☐ | Dual use research of concern |
| ☒ | ☐ | Plants |

## Methods

| n/a | Involved in the study | |
|---|---|---|
| ☒ | ☐ | ChIP-seq |
| ☒ | ☐ | Flow cytometry |
| ☒ | ☐ | MRI-based neuroimaging |

# Clinical data

Policy information about clinical studies

All manuscripts should comply with the ICMJE guidelines for publication of clinical research and a completed CONSORT checklist must be included with all submissions.

| | |
|---|---|
| Clinical trial registration | n/a |
| Study protocol | n/a |
| Data collection | No data was collected as part of this study. The used data is described in detail in https://pubs.rsna.org/doi/10.1148/ryai.220047. |
| Outcomes | n/a |
